# Assessing Usefulness of the Dashboard Instrument to Review Equity (DIRE) Checklist to Evaluate Equity in Public Health Dashboards: Reliability Study

**DOI:** 10.2196/71094

**Published:** 2025-12-04

**Authors:** Paulina Sosa, Emir A Syailendra, Harold P Lehmann, Hadi Kharrazi

**Affiliations:** 1 Department of Health Policy and Management, Bloomberg School of Public Health, Johns Hopkins University, 615 N Wolfe St, Baltimore, MD, 21205, United States, 1 202 379 8940; 2 Section of Biomedical Informatics and Data Science, School of Medicine, Johns Hopkins University, Baltimore, MD, United States

**Keywords:** public health, dashboards, equity, COVID-19, informatics, public health informatics, public health data, decision support

## Abstract

**Background:**

The COVID-19 pandemic was a critical time for public health, and though dashboards remained a source of critical health information for decision-makers, key gaps in equity-based decision support were revealed. The DIRE (Dashboard Instrument to Review Equity) Framework and Checklist tool was developed to be a practical tool for public health departments to use in evaluating equity-based decision support mechanisms in their dashboards.

**Objective:**

The objective of this agreement and reliability study was to validate the DIRE Checklist tool as a practical and reliable instrument for data practitioners to use in evaluating dashboards.

**Methods:**

This study was divided into 5 steps to conduct the necessary analysis for agreement and reliability. Step 1 completed the development of the DIRE Checklist tool in Qualtrics (Qualtrics International Inc). Step 2 focused on the parameters required for the selection of the 26 US state–based dashboards. Step 3 was the user testing and assessment process during which each reviewer applied the DIRE tool to each dashboard. Step 4 involved conducting different assessment methods to specifically calculate the comparative analysis, interrater agreement, intraclass correlation coefficients, and the cosine similarity for the Qualtrics, reviewer, and categorical scores. Finally, Step 5 involved conducting any qualitative assessment required on the notes.

**Results:**

A total of 26 dashboards were evaluated using the DIRE Checklist tool by 2 reviewers. The overall percentage comparison for the Qualtrics Score was 31.7% (28.24/89) for Reviewer 1 and 41.8% (37.16/89) for Reviewer 2, resulting in a relative percent agreement of 72.7%. Additionally, the categorical scores showed substantial to high agreement across most categories based on percent agreement within each category. The intraclass correlation coefficient scores indicated varying levels of agreement across different categories, with good agreement observed for the Qualtrics score.

**Conclusions:**

The reliability and agreement result of the study confirmed strong performance of the DIRE checklist tool. The scores calculated were evaluated consistently and reliably by both raters—demonstrating the DIRE Checklist tool’s ability to robustly evaluate different dashboards across a number of different categories and parameters.

## Introduction

The COVID-19 pandemic provided a pivotal juncture to assess the reliability and effectiveness of traditional public health practices [1]. Between 2020 and 2023, public health departments, decision-makers, and data practitioners were faced with time-sensitive information requiring urgent decisions to be made. Public health dashboards played a key role in the decisions made by public health officials [2]; however, shortcomings in the design and implementation of these dashboards created challenges in decision-making, thus increasing the possibility of unintentional and undesired outcomes [3,4]. Specifically, while dashboards were the focal source for many of these critical decisions [5], several gaps in data and information limited their effectiveness as a tool for decision support [6].

Several studies have confirmed that minority and vulnerable populations in the United States faced higher rates of hospitalization and death during the COVID-19 pandemic, but these populations (eg, Black communities, Hispanic communities, and Indigenous communities) continued to receive less funding, vaccination opportunities, and health care access [7-10]. The evidence of public health inequities during the COVID-19 pandemic has further supported the efforts to integrate and communicate such health disparity information in public health dashboards.

Incorporating basic health equity information (eg, stratification of outcomes by demographics and geography) in public health dashboards has been shown to be key in communicating potential disparities across population groups and communities [11]. Additionally, to more cohesively address equity gaps, data sources representing social determinants of health (SDOH), also referred to as social and structural drivers of health, could provide additional equity dimensions (eg, housing, education, income, and employment) for public health decision-makers to address the needs of communities affected by health inequities [12]. Previous studies have confirmed the need for data practitioners to integrate data representing these gaps into public health dashboards [12].

To address the integration of health equity data and information in public health dashboards, in a previous study, the Dashboard Instrument to Review Equity (DIRE) framework was developed and refined [11]. The refinement of the DIRE framework was accomplished by collecting quantitative and qualitative data from public health data practitioners and decision-makers, which was [11] used to develop a user-friendly checklist (the DIRE checklist) that can be used by data practitioners to apply the DIRE framework principles to their public health dashboards.

The DIRE checklist gives data practitioners and developers a streamlined and useful tool to ensure that equity-based data and decision support guidelines are included and added to public health dashboards. Despite these promising potential outcomes, previous studies have not assessed the usability and reliability [13] of the DIRE checklist when applied by different users to real-world public health dashboards (eg, COVID-19 dashboards). Once reliability and usability are proven, a tool like DIRE could transform the preparedness of dashboards, visualizations, and potentially decision support for future emergencies.

This study aims to assess the reliability of the DIRE checklist as a practical instrument for data practitioners to assess the integration of health equity information in their own dashboards [13]. To achieve this, we applied the DIRE checklist to available US COVID-19 and respiratory dashboards and evaluated its reliability through 2 independent raters while also identifying general areas for improvement. In choosing dashboards for evaluation, we constructed a representative set of state-based dashboards, enabling us to provide a qualitative assessment of the current state of dashboards in incorporating health equity information. The findings of this study could be used to unveil common gaps in COVID-19 dashboards in representing health equity information for decision-making purposes. The DIRE checklist provides data practitioners with an approach to implement equity-based data guidelines into their dashboards to help decision-makers identify timely trade-offs to prevent unintended inequities during similar future emergencies.

## Methods

### The DIRE Checklist

In our previous studies, the checklist was adapted to directly reflect the DIRE framework components, resulting in 17 questions—recording the presence of six high-level DIRE framework categories: (1) data availability and sources, (2) visualizations and analyses, (3) human-computer interface (HCI), (4) decision support visualizations, (5) equity-based decision support, and (6) community interventions (Multimedia Appendix 1). Upon adaptation from the framework, the DIRE checklist underwent a series of internal reviews and edits by several users with varying backgrounds in public health and dashboard development [11].

Upon completion of the DIRE checklist design and development, the checklist was implemented in Qualtrics (Qualtrics International Inc [14]), a web-based survey administration software, for ease of use and scoring. The DIRE checklist is specifically built to be used by data practitioners, regardless of their public health background. The checklist gives data practitioners an easy-to-use list of questions to assess if their dashboards meet the DIRE criteria [11] (Multimedia Appendix 2). The study team adhered to the GRRAS (Guidelines for Reporting Reliability and Agreement Studies) in conducting the study and reporting the results [13].

### US State-Based Dashboard Selection

Several state-based COVID-19 and respiratory disease dashboards were selected using the purposive sampling technique from across the United States in 2024 [15]. This approach allowed the deliberate selection of dashboards that reflected diversity in key characteristics relevant to the assessment process and analysis thereafter. Dashboards were selected based on several diversity parameters to ensure that the testing of the checklist was applied across different demographics of US states. Dashboards were selected based on diversity and representation of the following parameters: (1) population size, based on the US Census Bureau [16] (ie, large, medium, or small); (2) demographic diversity, based on Census ACS (American Community Survey) 2019 estimates [17] (eg, ethnicity, race, and age); (3) median gross domestic product (GDP) and income of the state [18]; and (4) geographic diversity as defined by the Bureau of Economic Analysis (BEA) Regions (ie, Far West, Great Lakes, Mideast, New England, Plains, Rocky Mountain, Southeast, Southwest, and Territories). By using these criteria, we ensured that our sample represented a wide range of state demographics for more comprehensive testing of the DIRE checklist’s health equity measures. Selection of the state-based dashboards was not planned as a factorial design, but as one of overlapping DIRE categories; thus, US-related diversity attributes were represented.

Upon categorization of states by these parameters, we chose a selection of at least 1‐3 dashboards per region and territory, based on the inclusion of all categories and upon the dashboard being (1) available for public viewing and (2) fitting the definition of a dashboard, “a visual display of the most important information needed to achieve one or more objectives, consolidated and arranged on a single screen so the information can be monitored at a glance*”* [5]. Upon completion of this process, the top 26 dashboards that satisfied these parameters were selected for user testing and application of the DIRE checklist (Multimedia Appendix 3).

### User Testing and Assessment Process

The study consisted of 2 test users: Reviewer 1 (PS) and Reviewer 2 (EAS). Both reviewers applied the DIRE checklist to the 26 state dashboards to showcase the average “score” of dashboards against the categories of the DIRE framework and to test the checklist’s reliability. Each reviewer was provided with the same set of instructions, a list of selected dashboards, and a Qualtrics link for the DIRE checklist to conduct the assessment. This process aimed to (1) evaluate the ease of use of the checklist by assessing the time required to complete it, (2) examine the checklist’s reliability by measuring interrater agreement when applied by 2 different users on the same dashboard, (3) evaluate the validity of the tool in measuring what it was built to measure, and (4) assess the general state of currently available US COVID-19 dashboards and whether they address DIRE framework elements.

The primary objective of applying the checklist to the dashboards was to assess the level of agreement between the 2 reviewers. This process not only evaluated the consistency of their evaluations but also confirmed the checklist’s clarity and comprehensibility between 2 reviewers with different backgrounds and expertise. By comparing their assessments, we aimed to ensure that both reviewers interpreted the checklist in the same way, thereby confirming its effectiveness as a reliable evaluation tool. Inherently, the validity of the tool was also assessed in this process by confirming whether it measured the components of the DIRE framework and health equity that it was meant to measure.

Dashboard identifiers (eg, states and target population) were replaced in the analysis via a code to avoid possible reviewer bias in the analysis. Each reviewer followed a standard operating procedure (Multimedia Appendix 4) to ensure consistency of methods, conditions, and parameters and to mitigate potential deviations or biases in use. Each reviewer used a shared spreadsheet to track dashboard assessment times, scores, and any relevant observation notes. Each reviewer tracked time, at the start and close of each assessment, to calculate the average time needed to use the checklist. Each reviewer took notes to assess the usability, efficiency, validity, and reliability of the checklist. Additionally, each reviewer used the Qualtrics-based DIRE checklist to mark and submit the present parameters observed in each dashboard, which would automatically calculate the total DIRE checklist score. Finally, each reviewer provided an overall grade for the dashboard using a subjective approach. This process resulted in both quantitative and qualitative data.

### Assessment Analysis

#### Data Collection and Organization

Completed checklists of both users were consolidated and organized into one spreadsheet for comparison and analysis. Raw data collected in Qualtrics from each of the completed checklists were downloaded into a unified spreadsheet. The data were then organized in the spreadsheet for analysis in Tableau (version 2024.2.2; Tableau Software LLC, Salesforce [19]) and R (version 4.2.1; R Foundation for Statistical Computing [20]).

#### Scoring and Comparative Analysis

Multiple scores were generated using the DIRE checklist for comparative analysis. First, each of the DIRE framework categories and subcategories was mapped to DIRE checklist questions to evaluate relevant aspects of the dashboard. Each category was then analyzed as a whole or as a subcategory by the reviewer (ie, the categorical and subcategorical scores). Second, 2 overall scores were calculated after applying the DIRE checklist. One score was done quantitatively by Qualtrics as the sum of scores captured by all checklist questions (ie, the DIRE checklist or Qualtrics score). The other score was generated qualitatively using the reviewer’s subjective assessment of the availability of DIRE attributes and user preferences in navigating the dashboard (ie, the Reviewer score or grade).

#### Interrater Agreement Measurement

The primary goal of the interrater calculation was to determine the degree to which both reviewers provided consistent ratings when applying the checklist to the same dashboard. High agreement levels would indicate whether the checklist was interpreted similarly by both reviewers across dashboards, which would reinforce the checklist’s reliability. Interrater agreement was calculated using several methods to assess the consistency of the DIRE checklist scores, categorical scores, and textual notes between the 2 reviewers. The percentage agreement was also calculated for the reviewer score; however, the subjective nature of this score limited its interpretation. The methods used to measure interrater agreement were (1) percent agreement, (2) intraclass correlation coefficients (ICCs), and (3) cosine similarity.

The comparison of percentage agreement was conducted to quantify and assess the level of agreement between the 2 reviewers for the DIRE checklist and categorical scores. For the first comparison, the average DIRE checklist score per dashboard was divided by the total number of dashboards to yield the percentage of DIRE checklist score by reviewer. For the second comparison, the average categorical score per dashboard (ie, the sum of all subcategories divided by the total possible sum) was divided by the total number of subcategories to yield the categorical score percentage by reviewer. Each of these percentages was then compared between the 2 reviewers to evaluate how closely their assessments aligned. This approach provided a general measure of interrater agreement in the DIRE checklist and categorical scores.

To assess the consistency of the average percentages for each score, the percentage agreement was calculated by comparing the difference between their score percentages to the average of those score percentages. A validated formula [21] was applied to the DIRE checklist and categorical scores across all dashboards to quantify the level of agreement between reviewers. The thresholds that were used to interpret results were based on the threshold recommendations provided by McHugh [21].

Agreement between the 2 reviewers on continuous outcomes was analyzed using the ICC3 and ICC3k approaches [22]. The ICC measure is ideal for scenarios with 2 independent raters evaluating the same participants. ICC accounts for both consistency and absolute agreement in ratings, making it a suitable methodology for evaluating how similarly the 2 reviewers assessed the outcomes. The ICC calculation was performed using R version 4.2.1 [20]. Thresholds used to interpret results were based on the threshold recommendations provided by Bartko [22].

The semantic similarity of the reviewers’ textual notes was assessed using the cosine similarity measurement of their vector embeddings. To obtain meaningful representations, we used the all-mpnet-base-v2 Transformer-based model [23], a state-of-the-art sentence embedding model designed to capture deep semantic meanings within text. The model produces dense vector embeddings that effectively capture the nuances of language, allowing the analysis of subtle semantic differences and assessment of the textual agreement between the reviewers. The cosine similarity values were then converted into angles to provide a more intuitive understanding of alignment [24]. Semantic similarity analysis was accomplished using Python 3.12 (Python Software Foundation [25]).

#### Descriptive Analyses

After calculating the interrater agreement, the reviewers conducted further descriptive analysis to gauge the performance of the dashboards using the checklist. The following descriptive statistics were calculated to summarize the data: average, median, SD, and 2 potential baseline thresholds (ie, an ideal baseline based on the total possible score and a realistic baseline based on the median). Fixed base tertiles were also calculated to categorize the average total percentage between both reviewers across 26 dashboards. Dashboards were organized into 3 tertiles: low (tertile 1: 0%‐33.3%), medium (tertile 2: 33.3%‐67%), and high (tertile 3: 67%‐100%). Deciles were also calculated to visualize the spread of dashboard scores on a map. Descriptive analyses were conducted using the DIRE checklist, categorical, and reviewer scores.

### Ethical Considerations

This study was reviewed and deemed exempt by the Johns Hopkins School of Public Health’s Institutional Review Board (IRB00018696). This study analyzed publicly available dashboards and did not involve human participants or private health information. No individual-level protected health information was collected in this study.

## Results

### Interrater Agreement

The overall percentage of the DIRE checklist score was 31.7% (28.24/89) for PS and 41.8% (37.16/89) for ES. To assess the consistency of scores between the 2 reviewers, percent agreement was calculated between their 2 average percentages, resulting in a percent agreement of 72.7% (1-(|41.8-31.7|/(31.7+41.8/2)); Multimedia Appendix 5) [21].

**Figure 1.**
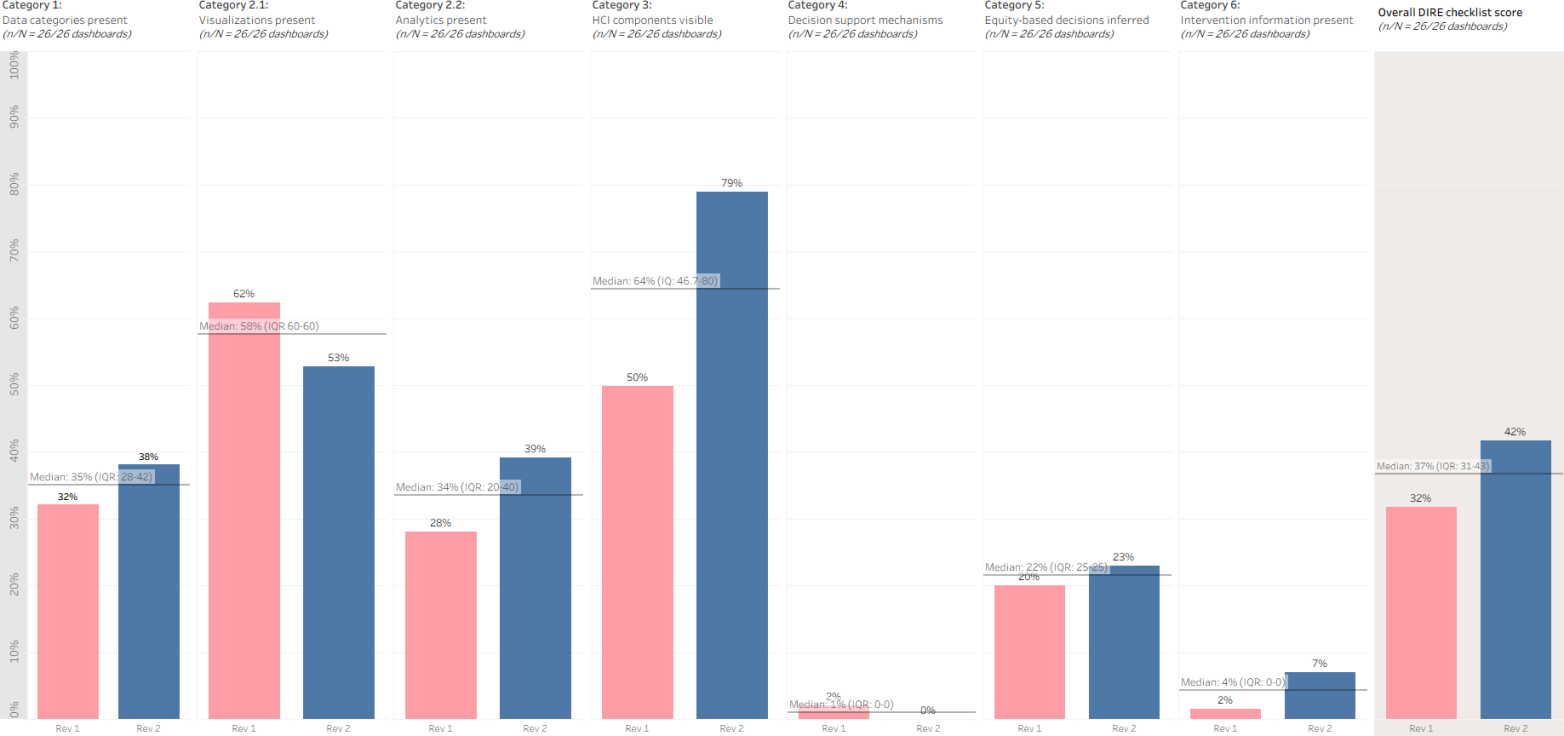
Overall comparison of category and DIRE (Dashboard Instrument to Review Equity) checklist scores across reviewers (N=26 dashboards). HCI: human-computer interface; Rev: Reviewer.

The percentage comparison of the categorical score of each reviewer was applied by calculating the percentage comparison for each of the 6 DIRE framework’s categories. These comparisons yielded varying levels of agreement across the 6 categories. For example, the percentage comparison for category 1 was 32.1% (96.4/3) for PS and 38.1% (114.4/3) for ES, while category 2.1 showed scores of 62.4% (78/125) and 52.8% (66/125), respectively (Figure 1).

DIRE checklist categories 1 (data representation), 2.1 (visualizations), 2.2 (analyses), 3 (HCI components), and 5 (equity-based decisions) showed moderate to substantial percent agreement. Categories 4 (decision support) and 6 (interventions) were both graded too low to compute an accurate percent agreement; however, both reviewers rated these categories as extremely low (Table 1).

**Table 1.** Overall percentage comparison, percentage agreements, and agreement levels.

DIRE^a^ category	Category name	Reviewer 1, n/N (%)	Reviewer 2, n/N (%)	Percent agreement (%)^b^	Agreement level [21]
Categories 1‐6	DIRE checklist score	28.24/89 (31.7)	37.16/89 (41.8)	72.72	Substantial
Category 1	Data representation	96.4/ 3 (32.1)	114.4/3 (38.1)	82.9	High
Category 2.1	Visualizations	78/125 (62.4)	66/125 (52.8)	83.3	High
Category 2.2	Analyses	35/125 (28)	49/125 (39.2)	66.7	Substantial
Category 3	HCI^c^ components	187/375 (49.9)	296/375 (78.9)	54.9	Moderate
Category 4	Decision support	1/50 (2)	0/50 (0)	–100	High
Category 5	Equity-based decisions	20/100 (20)	23/100 (23)	86	High
Category 6	Interventions	4/253 (1.6)	18/253 (7.1)	–27.3	High

a DIRE: Dashboard Instrument to Review Equity.

b Formula: 1-(|PS%- ES%|/(ES%+PS%/2.

c HCI: human-computer interface.

ICC results showed varying levels of agreement across different categories, with moderate to good agreement in the DIRE Qualtrics score (ICC3=0.47; ICC3k=0.64) and category 1 (data representation; ICC3=0.59; ICC3k=0.74), indicating consistency between the 2 reviewers. In contrast, categories 2 (visualizations), 3 (HCI components), and 4 (decision support) showed slight agreement (ICC3=0; ICC3k=0) due to low variation in scores between the 2 reviewers, making it difficult for the ICC to capture any meaningful agreement. Notably, categories 5 (equity-based decisions) and 6 (interventions) showed fair to moderate agreements, with ICC values ranging between 0.34 and 0.57 (Table 2).

**Table 2.** Intraclass correlation cooefficient calculations for the Dashboard Instrument to Review Equity checklist and category scores.

DIRE^a^ category and type		ICC^b^ (95% CI)	*P* value	Agreement
DIRE checklist score (overall)				
ICC3		0.47 (0.10 to 0.73)	.007	Moderate
ICC3k		0.64 (0.19 to 0.84)	.007	Moderate
1 - Data representation				
ICC3		0.59 (0.26 to 0.80)	<.001	Moderate
ICC3k		0.74 (0.41 to 0.89)	<.001	Good
2.1 - Visualization				
ICC3		0 (–0.39 to 0.39)	.50	Slight
ICC3k		0 (–1.27 to 0.56)	.50	Slight
2.2 - Analyses				
ICC3		0.25 (–0.15 to 0.58)	.11	Fair
ICC3k		0.40 (–0.36 to 0.74)	.11	Fair
3 - HCI representation				
ICC3		0.00 (–0.39 to 0.39)	.50	Slight
ICC3k		0.00 (–1.27 to 0.56)	.50	Slight
4 - Decision support				
ICC3		0.00 (–0.39 to 0.30)	.50	Slight
ICC3k		0.00 (–1.27 to 0.56)	.50	Slight
5 - Equity-based decisions				
ICC3		0.34 (–0.06 to 0.64)	.05	Fair
ICC3k		0.50 (–0.13 to 0.78)	.05	Moderate
6 - Interventions				
ICC3		0.40 (0.02 to 0.69)	.02	Fair
ICC3k		0.57 (0.04 to 0.81)	.02	Moderate

a DIRE: Dashboard Instrument to Review Equity.

b ICC: intraclass correlation coefficient.

The observed pattern of the cosine results highlighted varying degrees of semantic agreement between the notes. The highest cosine similarity was observed for dashboard 9 (0.96), indicating a strong alignment between the 2 reviewers’ notes. Dashboard 2 (0.69), 13 (0.67), and 26 (0.66) also exhibited moderate similarity levels, suggesting that the notes share some common features, but with more variation. Other dashboards showed lower similarities between the reviewers’ notes (Table 3).

**Table 3.** Cosine similarity of reviewer notes across dashboards (N=26).

Rank	Dashboard number	Cosine similarity
1	9	0.96
2	2	0.69
3	13	0.68
4	26	0.67
5	10	0.64
6	16	0.62
7	3	0.61
8	4	0.6
9	11	0.59
10	1	0.58
11	23	0.57
12	6	0.56
13	22	0.53
14	15	0.52
15	20	0.49
16	5	0.49
17	8	0.48
18	14	0.45
19	25	0.44
20	21	0.42
21	18	0.42
22	17	0.42
23	24	0.39
24	7	0.38
25	12	0.34
26	19	0.31

### Descriptive Statistics

DIRE categories 1 (data), 2 (visualization and analytics), and 3 (HCI components) were rated comparably high by the 2 reviewers. For category 1, reviewers agreed that clinical data are more frequently present than social or environmental data (Figure S1 in Multimedia Appendix 5). This category is where data sources reflecting different types of SDOH would typically appear, which is an area directly aligned with the DIRE checklist. For category 2, both reviewers agreed that variations of visualization techniques (ie, maps, graphs, and charts) were more prominent than analytic methods (eg, descriptive and predictive). Indeed, most dashboards only displayed descriptive analyses (Figure S2 in Multimedia Appendix 5). For category 3, despite some disagreements, both reviewers agreed that dashboards were at most moderately showcasing HCI components. Reviewers agreed that certain HCI components were more visible than others, including feedback and visual cues, system flexibility, consistency, and simplicity (Figure S3 in Multimedia Appendix 5).

In contrast to categories 1-3, DIRE categories 4 (decision support), 5 (equity decisions), and 6 (interventions) were rated low across the dashboards. For category 4, reviewers agreed that all dashboards were lacking any form of decision support (Figure S4 in Multimedia Appendix 5). For category 5, both reviewers agreed that very few dashboards showed any form of equity-based decision-making. However, based on the demographic data presented, Reviewer 1 denoted 20 dashboards, and Reviewer 2 denoted 19 dashboards presenting visualizations where equity-based decisions could potentially be inferred (Figure S5 in Multimedia Appendix 5). For category 6, both reviewers agreed that all dashboards were lacking detailed information on interventions (Figure S6 in Multimedia Appendix 5).

The average DIRE checklist score was calculated as 37% (ie, 32.7 out of a total possible score of 89) when measured across all dashboards. The average reviewer score was 9.24 out of the highest grade of 13. More specifically, the median reviewer score for PS was 9 (IQR 7-10) and for ES was 11 (IQR 8-12), as illustrated in Figure S7 in Multimedia Appendix 5.

Baseline thresholds (ie, both ideal and realistic) were calculated for each score (ie, DIRE checklist, category, and reviewer). The ideal baseline is defined at 80% [21,26]; however, none of the scores reached this threshold. Thus, baselines were developed based on the median scores to provide a more realistic baseline of current dashboard expectations. The median-derived realistic baseline for category 1 was calculated at 35.1% (IQR 32.1%-38.1%), category 2.1 at 57.6% (IQR 52.8%-62.4%), category 2.2 at 33.6% (IQR 28%-39.2%), and category 3 at 64.4% (IQR 49.9%-78.9%). Categories 4-6 were scored too low to develop a relevant baseline (Figure 1). The overall DIRE checklist score’s realistic baseline was 37% (Figure 1).

**Figure 2.**
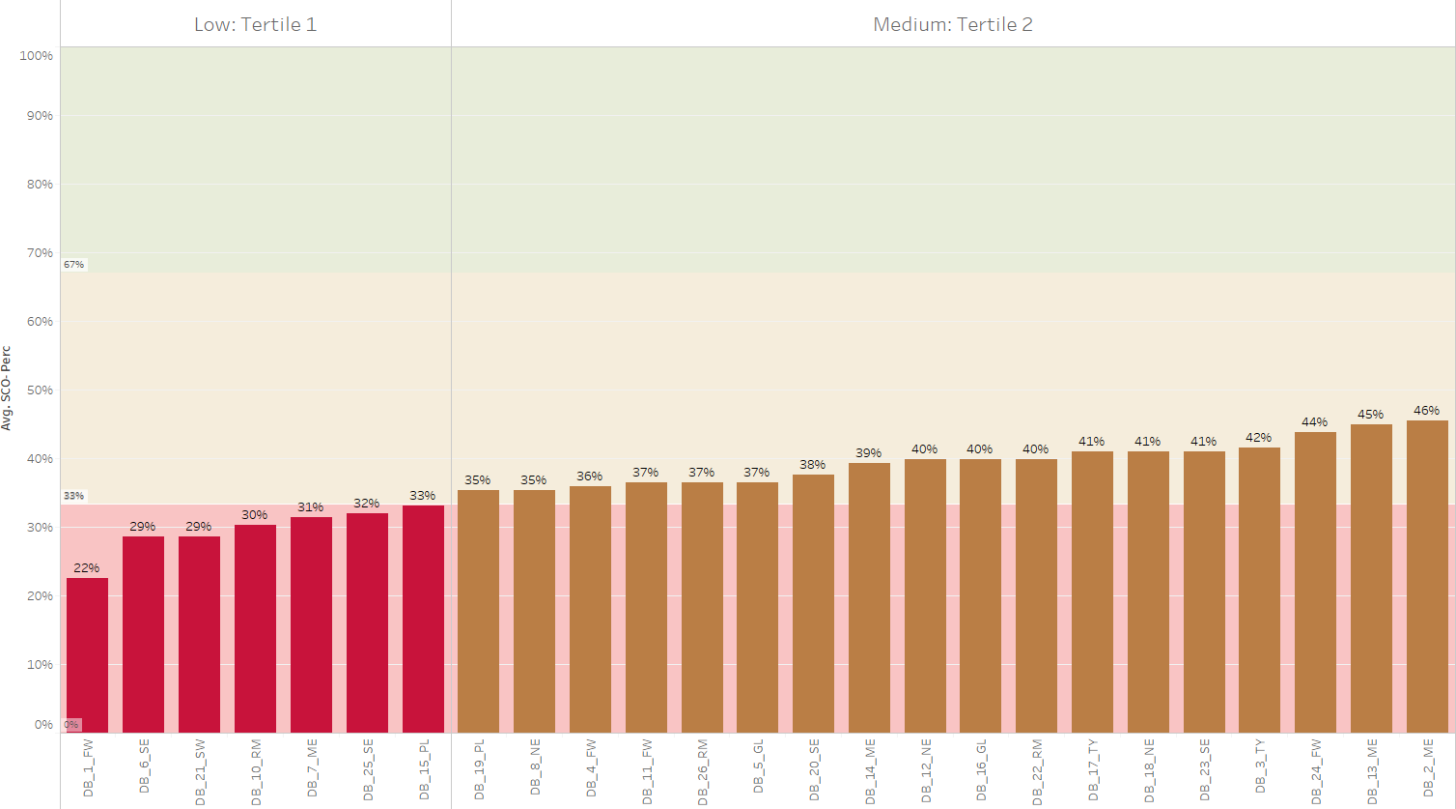
Average total scores by dashboard and tertile. FW: Far West; GL: Great Lakes; ME: Mideast; NE: New England; PL: Plains; RM: Rocky Mountain; SE: Southeast; SW: Southwest; TY: Territory.

**Figure 3.**
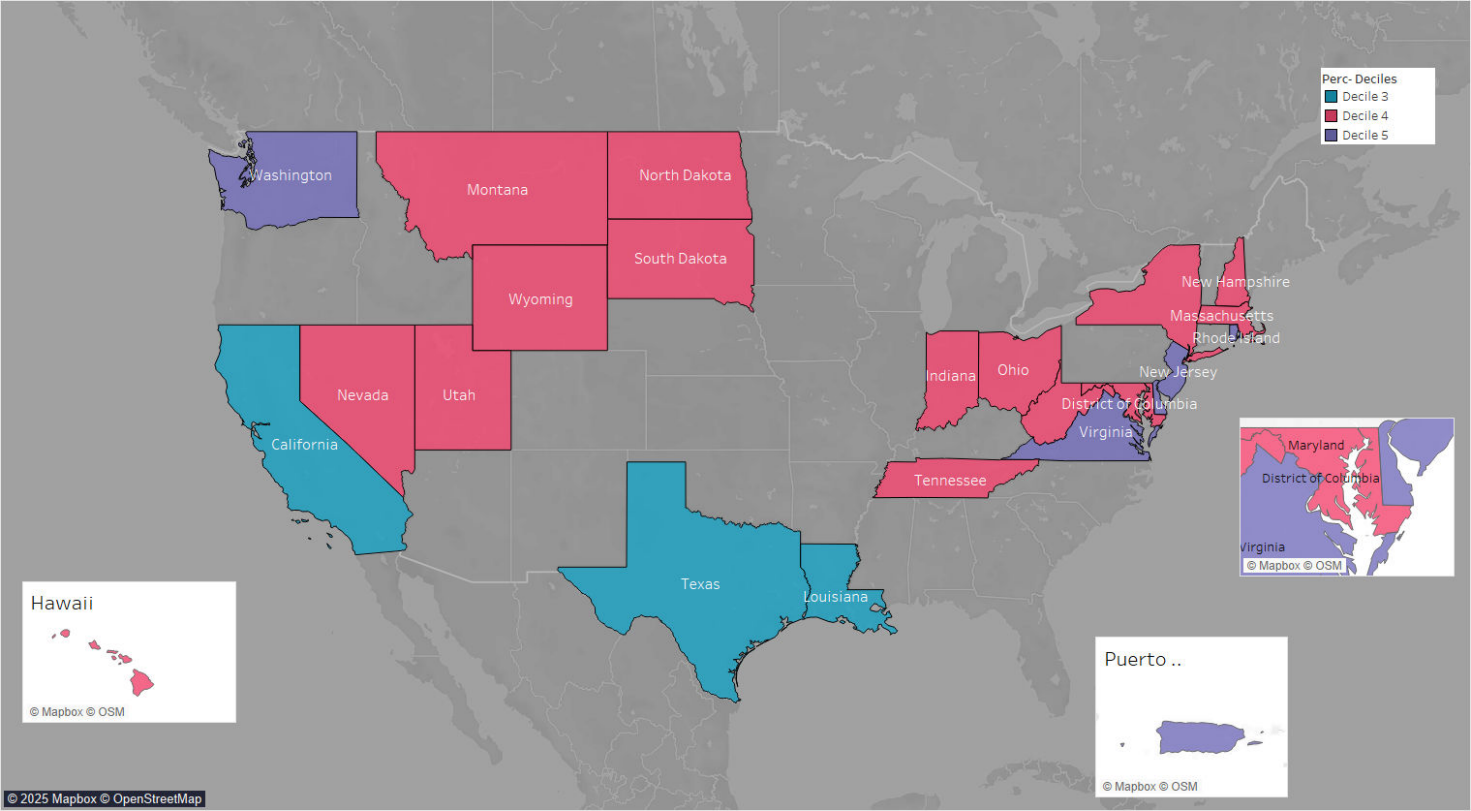
Average total scores by dashboard, state, and decile.

Seven state-based dashboards were scored in the low tertile (tertile 1: 0%‐33.3%) while 18 dashboards were placed in the medium tertile (tertile 2: 33.3%‐67%). None of the dashboards scored high enough to reach the high tertile (tertile 3: 67%‐100%), so hence the highest tertile is not shown in Figure 2. Dashboards were visualized on the US map to depict the spread by decile. Three dashboards ranged in decile 3 (20%‐30%), 15 dashboards fell in decile 4 (31%‐40%), and 7 dashboards fell in decile 5 (41%‐50%; Figure 3). No state-based dashboards ranged above decile 5, with the highest score at 45.5%. Multimedia Appendix 6 includes the full list of dashboards, states, and the total average percent scores.

**Figure 4.**
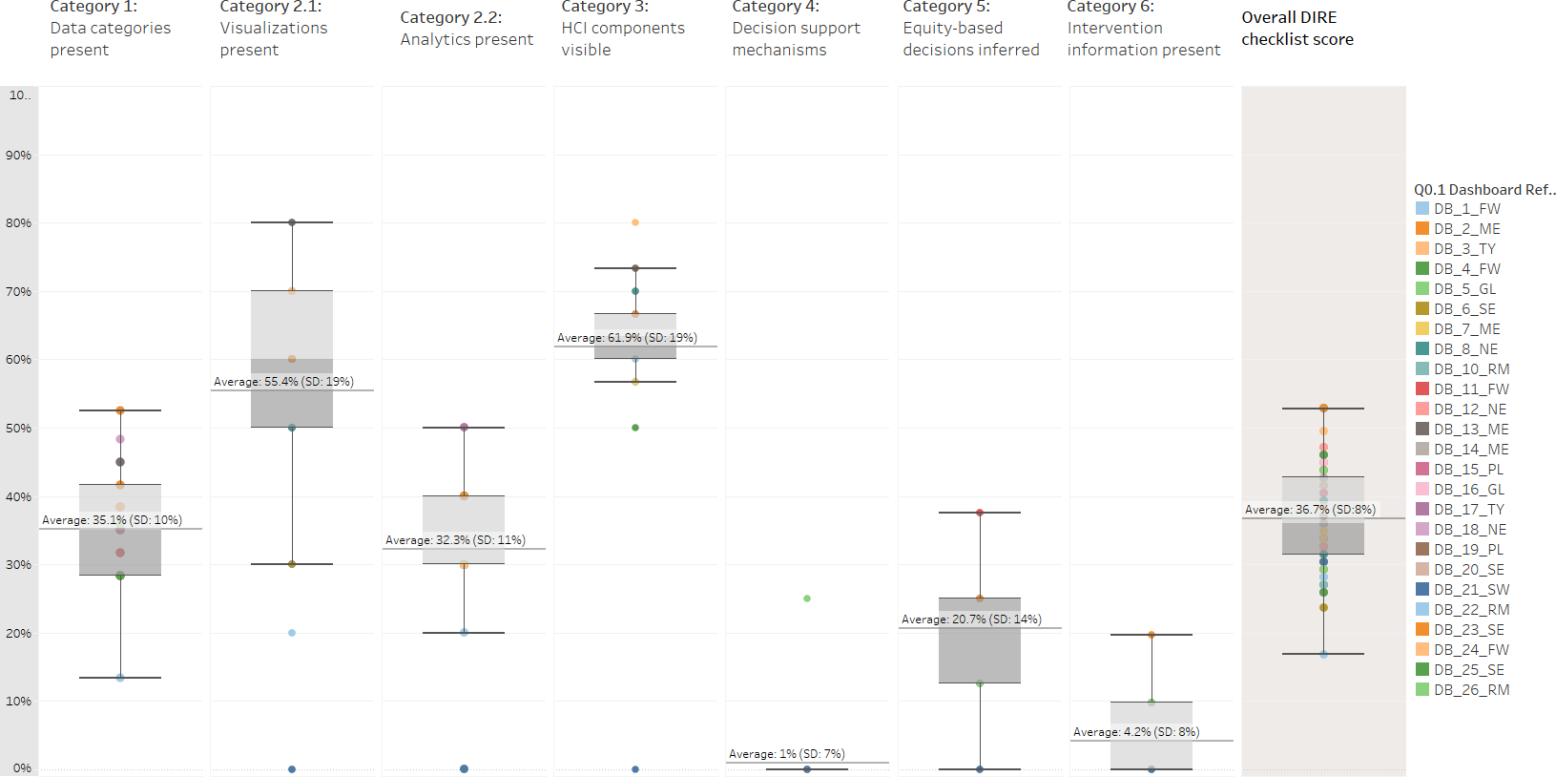
Boxplot of DIRE (Dashboard Instrument to Review Equity) checklist and category scores (N=26 dashboards). FW: Far West; GL: Great Lakes; ME: Mideast; NE: New England; PL: Plains; RM: Rocky Mountain; SE: Southeast; SW: Southwest; TY: Territory.

Category and DIRE checklist scores were visualized using boxplots (Figure 4). The boxplots displayed the range of scores, outliers, and the average for each score. Certain categories scored higher (eg, visualizations and HCI components), while other categories scored very low (eg, decision support mechanisms and intervention).

Comments entered by the reviewers were explored for common themes; however, due to the limited textual data, a full thematic analysis was not feasible (Multimedia Appendix 7).

## Discussion

### The DIRE Checklist

The goal of this study was to refine and launch the DIRE checklist, a user-friendly decision-support tool designed to help data practitioners and dashboard developers integrate health equity into public health dashboards. While the primary goal was to assess the reliability and validity of the DIRE checklist, this study also highlighted common trends in the design and functionality of state-based COVID-19 dashboards.

### DIRE Checklist Performance and Review

#### DIRE Checklist Reliability and Validity

The interrater agreement results of the study confirmed the reliability of the DIRE checklist. The dashboards were evaluated consistently by both raters, demonstrating the checklist’s robustness. The percent agreement of 72.7% indicated a substantial level of agreement within the DIRE categories of data representation, visualizations, and equity-based decisions [21]. The results, however, also identified challenges in other categories where score variability was minimal. The ICC values showed “Moderate” agreement for the DIRE checklist score, indicating the overall consistency of the DIRE checklist. The variability found in certain categories for the ICC calculation reflects differences in reviewer perspectives but also suggests areas where the DIRE checklist may require further refinement. Additionally, minimal score variation in some categories was simply too difficult to calculate because no attributes were present for that category, yet the checklist effectively identified the presence or absence of these attributes. While this study focused on 26 out of more than 50 US states and territories, the purposive sampling that was used provides a diverse sampling of dashboards to establish reliability for the DIRE checklist. These findings underscore the importance of observing reviewer variability as part of the evaluation and application process and confirm the DIRE checklist’s capacity to support equity-based dashboard evaluation, while also considering whether certain refinements to the checklist could improve comparative results.

In this study, the validity of the DIRE checklist was confirmed via the application of the checklist. The process of application demonstrated whether intended parameters of health equity data representation in dashboards were present according to the categories of the DIRE framework. The checklist aligned with the DIRE framework and detected variability between dashboard content and context, thus confirming construct validity. The checklist also adequately covered key components of health equity and the DIRE framework in its assessment of dashboards, thus confirming content validity. Given this demonstrated validity of the checklist to determine whether DIRE categories were sufficiently addressed in the dashboard, the checklist can be used to strengthen and enhance the readiness of public health dashboards to support decision-makers with health equity data and decision support in public health emergencies and routine surveillance.

#### Implications for Public Health Departments

One of the key roles of dashboards is to “employ a visual medium for communication information… relevant for a concrete problem*.*” [5] Indeed, while dashboards are a critical part of communication, dashboards are also one of the most effective ways to communicate data for decision-making, compared to data reports or briefs. The reason for this is due to a dashboard’s ability to present relevant data that can visually provide information on inequities and to integrate real-time aspects of predictive and diagnostic tools to aid in decision support [27]. While several stakeholders are involved, decision support assists the decision-maker to make critical decisions that can support community efforts and interventions, leading to the anticipated result of a healthier community.

The reliability of the DIRE checklist as an equity-based evaluation tool supports its broader implications for public health dashboards. The study findings underscore the need for standardized equity assessment tools, like the DIRE checklist, in dashboard development to ensure a comprehensive evaluation of health disparities and inequities that disproportionately affect underserved communities [27]. As shown in Figures2^4^, most dashboards had an average total score of 31%‐60% (ie, medium tertile), with no dashboards scoring higher than an average of 45%. These results highlight the need for a sustainable and standardized tool to address health equity data and decision support in public health dashboards. Integrating the DIRE-based checklist in dashboard design and development could enable public health departments with equity-based decision support during public health emergencies.

A key realization from the findings is the need to improve access to multiple types of data sources that capture dimensions of SDOH to more effectively implement equity-based parameters at all levels of intervention. Collaboration among public health stakeholders is key to integrating DIRE-based metrics into public health dashboards and to better addressing the different types of inequities that affect communities [28]. Collaboration between health care and nontraditional stakeholders is critical to improve access to multiple types of data sources (eg, education and housing), which remains a significant challenge for data practitioners. Access to SDOH-related data sources remains limited, which constrains the comprehensive equity-based insights that the DIRE checklist encourages. By fostering a strong sense of partnership, stakeholders can revolutionize how different data are accessed, integrated, and used for empowering equity-based visualizations and decision support. The study findings confirm the need for strengthened collaboration between decision-makers and data practitioners to ensure meaningful progress in incorporating equity data in dashboards.

To promote equity considerations in public health resource allocations and interventions, policymakers could encourage equity-based evaluations for health care IT systems, including dashboards. This effort can enhance transparency and accountability in addressing health disparities [29,30]. Public health decision-makers might also consider encouraging equity-based evaluations using checklists, such as DIRE for public health systems and dashboards. Indeed, through the integration of the DIRE checklist, equity recommendations could more holistically be integrated into public health dashboards.

The findings of this study also revealed that the DIRE checklist is a practical tool to assess equity-based aspects of public health dashboards across different states. Figure 3 provides a snapshot of the DIRE checklist’s assessment of state-based dashboards across the United States and underscores the need for widespread improvement. While the DIRE checklist is not meant to rank dashboards, it is meant to help data practitioners and related stakeholders keep track of the data and information to include in their dashboards. In fact, the DIRE checklist should be used as a guide when building and integrating new health equity components during dashboard design and development. The DIRE checklist’s primary use case is to assist dashboard developers and data teams in public health departments and health departments to identify current and missing data available for dashboard integration. These stakeholders will also be able to identify potential barriers to certain missing data or information. Additionally, the checklist provides guidelines to ensure that dashboard design is considerate of other important equity components, including the incorporation of accessibility, diverse representation of demographic data, decision support visualizations, data transparency of sources and analyses, and integration of continuous user feedback to address disparities and evolving community needs [31,32].

### Evaluation and Insights of State-Based Dashboards

#### Positive Practice 1: Clinical, Demographic, and Geographic Data

This study showed that all 26 dashboards integrated clinical data sources. The most prevalent clinical data sources were electronic health records, hospital discharges, and data provided by other sources, such as laboratories. Examples of clinical variables included test results, hospital bed numbers, and percentage positivity for the disease being monitored. Findings also noted that over 80% (22/26) of dashboards included basic demographic data (ie, age, sex, and ethnicity). Over 88% (23/26) of dashboards included geographic data and visualization features (eg, maps) to highlight hotspots for required support. Although the availability of demographic and geographic data in dashboards is promising for health equity decision support, not all dashboards provided the option to stratify the clinical data, such as patient outcomes or health trends, using such data.

Public health dashboards tend to have simple descriptive visualizations and basic analyses to describe the current situation [33-35]. Although data representations are expanding in dashboards, gaps still exist in data systems used by the dashboards. With the DIRE checklist, we were able to disaggregate the types of data used in the dashboard. While it is a positive trend to see an increase in use of demographic and geographic data, this discovery underscores the need to identify solutions to remove barriers to data accessibility for other equity-relevant data sources, such as SDOH data (eg, housing, transportation, income, education, and employment) that would amplify equity in public health dashboards.

#### Positive Practice 2: Range of Dashboard Visualization Techniques

Results showed that at least 20 (out of 26) dashboards integrated maps, graphs, charts, and tables showcasing a wide range of visualization techniques. Different visualizations show different aspects of the data, thus providing an interactive approach to support decision-making regarding health equity challenges. Given the use of commercial visualization tools by many of the dashboards, most dashboards provided the same basic visualization features for each of the techniques (eg, most maps included a zoom option). Despite the increasing use of artificial intelligence in enhancing data visualization techniques [36,37], none of the reviewed dashboards incorporated such features to provide additional information on health equity challenges.

#### Positive Practice 3: HCI Components (User Interface Navigation)

To enhance the user experience and improve efficiency, developers have been motivated to integrate interactivity across pages and visualizations within their public health dashboards [38,39]. The majority of the reviewed state dashboards presented the following user interface components: (1) feedback cues and icons (ie, a progress bar as you scroll through the dashboard), (2) affordance or visual cues (ie, a clickable hyperlink underlined in blue), (3) flexibility of system (ie, interactive maps or graphs vs static images of data), and (4) visibility of components (ie, a “click here to filter” button that explains how it customizes the results or maps). These are critical attributes for user interface and underscore the considerable improvements across dashboard interface and interactivity in general. However, reviewers could not identify any dashboard that presented all possible HCI components (ie, 14 features) as listed in the DIRE checklist. Additionally, 10 of these HCI components were minimally, or not at all, integrated into the dashboards, which included features, such as error prevention, system accessibility, and system documentation of user actions. These additional components might require additional information, training, and direct support for developers, presenting a unique opportunity to strengthen future dashboards as a tool to support decision-making regarding public health equity.

#### Areas of Growth 1: Widespread Absence of Decision Support and Equity-Based Decisions

The study findings revealed that only 21.5% (5/26) of state dashboards incorporated equity-related information. Moreover, none of the dashboards incorporated equity-based decision support mechanisms. Indeed, none of the reviewed dashboards had any form of decision support built in, and only 4.4% (1/26) of the dashboards included general information on community-based interventions or related information. Results also revealed a general lack of comprehensive equity factors incorporated in existing public health dashboards. While some dashboards incorporated basic demographic stratification, they still failed to capture the multifaceted aspects of health equity adequately [27]. Previous studies have emphasized the importance of data disaggregation and visual representation in identifying disparities [27]. However, the study findings underscored the need for explicit equity-focused metrics and interpretability features to facilitate targeted interventions and policy decisions [27]. Overall, despite an increase in equity-based data and metrics in some of the reviewed dashboards, particularly concerning demographic data [40], more work is needed by state-based public health departments to implement the required adjustments to best address health equity needs of underserved communities in state and local dashboards.

The lack of equity-related decision support features in public health dashboards is a widespread issue [12] and raises 2 critical points of consideration. First, the widespread absence of decision support, equity-based decisions, and community intervention recommendations in all reviewed state-based dashboards raises concerns on whether these gaps reflect oversights by data practitioners or broader systemic issues within public health department operations. One possibility is whether public health departments inherently isolate decision support to a selective group of decision-makers, creating an internalized boundary or siloed approach, which excludes data practitioners. Alternatively, this issue may instead indicate a need within public health departments to collaborate more effectively with data practitioners to ensure they have the information required to integrate equity-based decision support components. Second, data practitioners may not have access to the data needed to develop these mechanisms. Data source fragmentation makes it difficult, if not impossible, to collect and use additional equity-based data required for decision-support visualizations and community interventions.

#### Areas of Growth 2: Innovatively Improving Data Accessibility (Including Social and Environmental)

The findings showed that many dashboards presented high-level social (eg, demographics) and environmental data (eg, geographical boundaries), but only 9 dashboards included census, survey, or economic data. Only one dashboard presented SDOH data, such as neighborhood or housing data, the social vulnerability index (SVI) [41], or assisted living residence data. These unique types of data sources are critical to elevating equity-based visualizations in dashboards, beyond current expectations but do also highlight the data access barrier faced by data practitioners. While data fragmentation is a well-known challenge, the DIRE checklist lists these variables to strengthen the type of equity-based decision support developers can integrate as innovatively as possible. Data fragmentation remains a significant challenge in public health data systems, requiring a national collaboration and solution to bridge the gap between data systems and increase communications between systems.

#### Areas of Growth 3: Expansion to Different Types of Analytics

Another finding highlighted the absence of certain analytics in dashboards, particularly the use of diagnostic (average of 12/26 dashboards), predictive (average of 2/26 dashboards), and prescriptive (0/26 dashboards) analytics. While the analytic methods used for reporting public health data have grown considerably, our results revealed gaps in providing such analytics in public health dashboards when addressing health equity data. First, despite the expansion of prescriptive and predictive analytics, especially in the wake of AI innovations [36,37,42], these analytics were not integrated into the dashboards. Second, despite the general acceptance of using traditional analytics for public health dashboards and decision support [43], none of the dashboards had leveraged the traditional analytical methods (eg, regression methods) to provide more insightful results regarding health equity. Given the additional training, resources, and support for data teams needed to integrate such analytical methods [44], it is important for data practitioners and public health departments to start integrating the use of these analytics and the supporting data sources, now rather than during a public health emergency. It is important to note that several barriers can play a role in a health department’s ability to provide certain data, analyses, or graphics, including capacity, budgets, and data accessibility.

### Limitations and Future Research

Despite the study results supporting the ease of use and reliability of the DIRE checklist in assessing the utility of public health dashboards for health equity decision-making, this study has limitations that should be considered when interpreting its results. First, the assessment of the DIRE checklist’s validity was limited to content and construct validities. Although the checklist is not considered a survey and should instead be treated as a guideline, future studies should further evaluate other dimensions of the checklist’s validity that may enhance its usability (eg, making the checklist shorter without losing any of the equity categories as defined by the DIRE framework). Second, the DIRE checklist’s reliability was assessed by calculating the interrater reliability measures between 2 reviewers. Additional prior knowledge and exposure to the tool may have affected the results of the study, even though this was mitigated through the operating procedure. Additional reviewers, especially with diverse backgrounds, technical expertise, and operational barriers, can increase the strength of the DIRE checklist’s reliability assessment. Third, qualitative results of the study (eg, textual notes, reviewer score, and grade) might have been affected by the inherent bias of the reviewers. Although this study mitigated bias as much as possible by clearly identifying the evaluation steps and instructions for notating observations, an inevitable level of bias is still built into this approach. This potential bias may have also affected quantitative results, specifically certain category scores (eg, HCI category showed high variability among the reviewers). This observation highlights the need to clarify the definition and requirements of each checklist item to mitigate any potential reviewer bias and individual interpretations. Fourth, this study contained a limited sample size of US-based COVID-19 dashboards (ie, 26 state dashboards, out of a total of 50 states and 16 territories), potentially impacting the generalizability of the study findings regarding the status of health equity data integration in other countries or non–COVID-19 dashboards.

To advance the DIRE checklist and strengthen the utility of public health dashboards for improving health equity during an emergency response, future studies could (1) apply and evaluate the checklist against a larger sample of dashboards representing more diverse populations and geographical boundaries (eg, city, county, and international dashboards), (2) conduct longitudinal studies to evaluate the long-term impact of the checklist on dashboard improvements in reducing health disparities and promoting equitable interventions and decisions, and (3) explore the development and validation of other technologies (which could include solutions, such as NLP technologies) to streamline and facilitate an easier use of the application of the DIRE checklist for dashboard developers.

### Conclusion

This study verified the reliability of the DIRE checklist by having 2 reviewers apply it to 26 US statewide COVID-19 dashboards. The results demonstrated the checklist’s strong reliability and its potential to assist data practitioners in integrating health equity and decision support into their future dashboards. The study also underscored key strengths and opportunities to strengthen the DIRE checklist for broader public use, while highlighting valuable insights on the current state of public health dashboards in health equity, decision support, and public health preparedness. The findings of this study suggest that most dashboards lack adequate integration of equity considerations, decision support, and community intervention recommendations, underscoring the need to continue strengthening these areas. The DIRE checklist offers a practical tool that can address these gaps, thus empowering public health departments to strengthen their dashboards for routine public health surveillance as well as address health equity challenges in future public health emergencies.

## Supplementary material

10.2196/71094 Multimedia Appendix 1 DIRE (Dashboard Instrument to Review Equity) framework.

10.2196/71094 Multimedia Appendix 2 DIRE (Dashboard Instrument to Review Equity) checklist tool.

10.2196/71094 Multimedia Appendix 3 Table of state dashboards used.

10.2196/71094 Multimedia Appendix 4 Dashboard review: standard operating procedure (SOP).

10.2196/71094 Multimedia Appendix 5 Analysis of results by category.

10.2196/71094 Multimedia Appendix 6 Average scores by dashboard and by quartile.

10.2196/71094 Multimedia Appendix 7 Thematic analysis.

## Abbreviations

ACS: American Community Survey
BEA: Bureau of Economic Analysis
DIRE: Dashboard Instrument to Review Equity
GDP: gross domestic product
GRRAS: Guidelines for Reporting Reliability and Agreement Studies
HCI: human-computer interface
ICC: intraclass correlation coefficient
SDOH: social determinants of health
SVI: Social Vulnerability Index
