# Supplementary material for: Assessing Usefulness of the Dashboard Instrument to Review Equity (DIRE) Checklist to Evaluate Equity in Public Health Dashboards: Reliability Study
Source: JMIR Public Health Surveill. 2025 Dec 4;11:e71094. doi: 10.2196/71094 (PMC12677865; doi:10.2196/71094)
Supplement: Multimedia Appendix 1 [file publichealth-v11-e71094-s001.pdf]

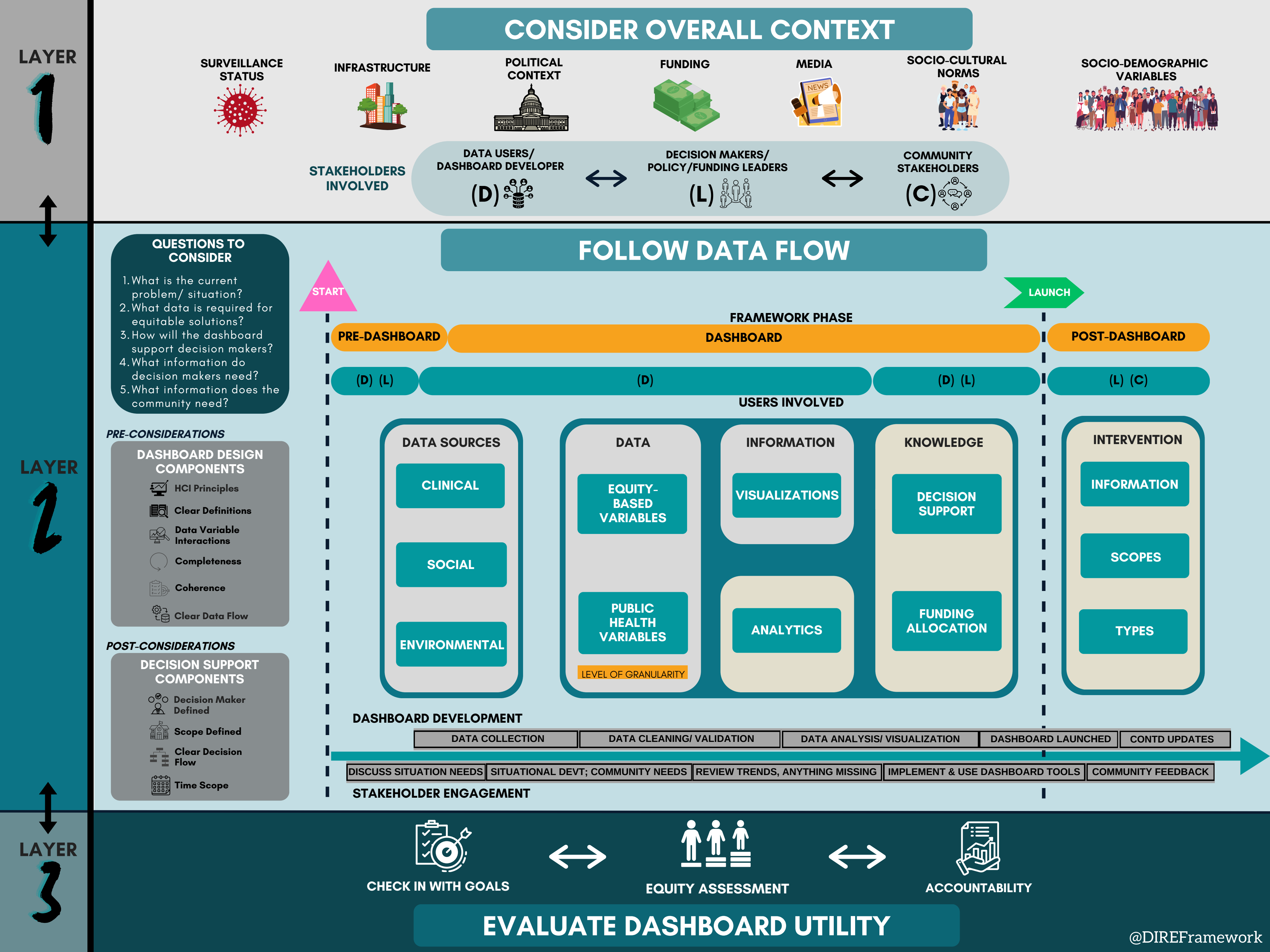
LAYER 1
CONSIDER OVERALL CONTEXT
SURVEILLANCE STATUS
INFRASTRUCTURE
POLITICAL CONTEXT
FUNDING
MEDIA
NEWS
SOCIO-CULTURAL NORMS
SOCIO-DEMOGRAPHIC VARIABLES
STAKEHOLDERS INVOLVED
DATA USERS/ DASHBOARD DEVELOPER (D)
DECISION MAKERS/ POLICY/FUNDING LEADERS (L)
COMMUNITY STAKEHOLDERS (C)
LAYER 2
FOLLOW DATA FLOW
QUESTIONS TO CONSIDER
1. What is the current problem/ situation?
2. What data is required for equitable solutions?
3. How will the dashboard support decision makers?
4. What information do decision makers need?
5. What information does the community need?
PRE-CONSIDERATIONS
DASHBOARD DESIGN COMPONENTS
HCI Principles
Clear Definitions
Data Variable Interactions
Completeness
Coherence
Clear Data Flow
POST-CONSIDERATIONS
DECISION SUPPORT COMPONENTS
Decision Maker Defined
Scope Defined
Clear Decision Flow
Time Scope
START
LAUNCH
FRAMEWORK PHASE
PRE-DASHBOARD
DASHBOARD
POST-DASHBOARD
(D) (L)
(D)
(D) (L)
(L) (C)
USERS INVOLVED
DATA SOURCES
CLINICAL
SOCIAL
ENVIRONMENTAL
DATA
EQUITY-BASED VARIABLES
PUBLIC HEALTH VARIABLES
LEVEL OF GRANULARITY
INFORMATION
VISUALIZATIONS
ANALYTICS
KNOWLEDGE
DECISION SUPPORT
FUNDING ALLOCATION
INTERVENTION
INFORMATION
SCOPES
TYPES
DASHBOARD DEVELOPMENT
DATA COLLECTION
DATA CLEANING/ VALIDATION
DATA ANALYSIS/ VISUALIZATION
DASHBOARD LAUNCHED
CONTD UPDATES
DISCUSS SITUATION NEEDS
SITUATIONAL DEVT; COMMUNITY NEEDS
REVIEW TRENDS, ANYTHING MISSING
IMPLEMENT & USE DASHBOARD TOOLS
COMMUNITY FEEDBACK
STAKEHOLDER ENGAGEMENT
LAYER 3
CHECK IN WITH GOALS
EQUITY ASSESSMENT
ACCOUNTABILITY
EVALUATE DASHBOARD UTILITY
@DIREFramework

DIRE Framework: Elements & Examples

DATA SOURCES	DATA	INFORMATION	KNOWLEDGE	INTERVENTION
**CLINICAL**	**EQUITY-BASED VARIABLES**<br>• Socio-demographic<br>• Economic<br>• Racial<br>• Other...	**VISUALIZATIONS**<br>• Maps<br>• Graphs<br>• Charts<br>• Tables<br>• Other...	**DECISION SUPPORT**<br>• DS Visualizations<br>• Decision Types<br>• Decision Trees<br>• Decision Paths<br>• Other...	**INFORMATION**<br>• Equity-based interventions<br>• Budget Trade-offs<br>• Community demographics<br>• Other...
**SOCIAL**	**PUBLIC HEALTH VARIABLES**<br>• Demographics<br>• Clinical<br>• Geographic<br>• Other...	**ANALYTICS**<br>• Descriptive<br>• Associational<br>• Causal<br>• Other...	**FUNDING ALLOCATION**<br>• Funding Sources<br>• Allocation Needs<br>• Resources Requests<br>• Community Needs<br>• Other...	**SCOPES**<br>• Schools<br>• Workplaces<br>• Restaurants<br>• Cities<br>• Other...
**ENVIRONMENTAL**				**TYPES**<br>• Vaccines<br>• Policies<br>• Funding<br>• Quarantine<br>• Other...
