# Supplementary material for: Assessing Usefulness of the Dashboard Instrument to Review Equity (DIRE) Checklist to Evaluate Equity in Public Health Dashboards: Reliability Study
Source: JMIR Public Health Surveill. 2025 Dec 4;11:e71094. doi: 10.2196/71094 (PMC12677865; doi:10.2196/71094)
Supplement: Multimedia Appendix 2 [file publichealth-v11-e71094-s002.pdf]

Dashboard Reference Number:

Reviewer Name

DIRE CHECKLIST - START

QUESTION 1. DATA VARIABLES

Does the dashboard present DATA VARIABLES or DATA TYPES from any of the following DATA SOURCES?
Select all that apply.

Yes, Clinical Data Sources (Hospital, Clinical, Lab, Vaccine, Testing, CDC, WHO)

Yes, Social Data Sources (Demographics, Census, American Community Survey (ACS), Economic)

Yes, Environmental Data Sources (Geographic, Household, Neighborhood, Transit)

No, None of these are presented.

IF YES TO CLINICAL, SOCIAL, OR ENVIRONMENTAL DATA SOURCES, REVIEW Q1.1, Q1.2, Q1.3.

IF NO, MOVE TO QUESTION 2.

Q1.1. Check off the CLINICAL data variables and/or sources you observe listed in the dashboard.
Select all that apply.

Hospital data (Data of the hospital including: # of beds taken, rates of admission, # of patients per doctor, etc...)

Clinical data (Data from Patient records including information about a patient's health status, i.e., patient diagnoses, demographics, etc...)

Electronic Health Record (EHR) / Electronic Medical Record (EMR) data (Data from a Health Information System for a clinician or hospital-- contains data of patient including test results, treatments, past medical history, etc...)

Laboratory data (Data on vaccines, tests, samples taken, etc...)

Other:

Q1.2. Check off the SOCIAL data variables and/or sources you observe listed in the dashboard.
Select all that apply.

Demographic data (Data regarding individuals focused on age, race, ethnicity, income, gender, marital status, etc...)

Census data (Data on population size, population characteristics-- tied to specific locations)

Survey data (Data from American Community Survey (ACS), National Health and Nutrition Examination Survey (NHANES), etc...)

Economic data (Data regarding neighborhoods/zip codes focused on income, employment, household, etc...)

Other:

Q1.3. Check off the ENVIRONMENTAL data variables and/or sources you observe listed in the dashboard.
Select all that apply.

Geographic data (Data on attributes or characteristics regarding the location of an area, neighborhood, zip code, region-- i.e., rural vs urban, population size, landmarks, markers, etc...)

Housing data (Data on households, family demographics of households, household-based median incomes, etc...)

Neighborhood data (Data on attributes or characteristics regarding the vicinity of a neighborhood or zip code-- i.e., Food deserts, Transit available, Closest clinics available, etc...)

Other:

QUESTION 2. DATA VISUALIZATIONS

Does my dashboard display VISUALIZATIONS and/or ANALYTICS?
Select all that apply.

Yes, visualizations are displayed (Maps, Graphs, Charts, Tables, Diagrams, etc)

Yes, analytics are displayed (Descriptive, Diagnostic, Predictive, Prescriptive, Real-Time, AI, etc)

No, None of these are displayed.

IF YES TO VISUALIZATIONS OR ANALYTICS, REVIEW Q2.1, Q2.2.

IF NO, MOVE TO QUESTION 3.

Q2.1. Does my dashboard display any of the following visualizations? *Select all that apply.*

Maps (Data displayed in a geographic-based map of area)

Graphs (Data displayed through a Line Graph, Bar Graph, 3D Graph, Area Graph)

Charts (Data displayed through a Pie Chart, Bubble Chart, Flowchart, Spider Charts, Gantt Chart)

Tables (Data displayed through an XY Table, Column Table- one grouping variable, Grouped Table- two grouping variables)

Diagrams (Data displayed through a Histogram, Venn Diagram, Radar, Scatter plot)

Other:

Q2.2. Does my dashboard provide any of the following analytics or analyses? *Select all that apply.*

Descriptive (Aggregate/Summarized data of "what happened", data trends/insights-- i.e. # of cases, # of deaths, # of hospitalizations)

Diagnostic (Data discovery, Data mining, "why it happened", i.e. # of neighborhoods with minimal access to vaccine clinics and correlated rate of high COVID cases

Predictive (Prediction of future trends, "what will happen", i.e. # of cases of COVID for next week, based on trend direction of last 4 weeks)

Prescriptive (Advanced analytics, Machine Learning, "what should we recommend", i.e. # of vaccines to be prepared to distribute in 3 months)

Real-Time analytics (Connected to a live data source/database, data updates in real time, "what is happening now", i.e. # of cases detected NOW vs LAST WEEK)

AI-based analytics (Use of artificial intelligence (AI) to automate processes, make insights, make predictions, recommendations)

Other:

QUESTION 3. HUMAN COMPUTER INTERFACE PRINCIPLES

Was the Dashboard easy to navigate and understand? *(Testing whether the dashboard integrates Human Computer Interface (HCI) principles to increase usability and readability?)*

Select all that apply.

1. FEEDBACK Cues/Icons (Does the dashboard have indications that a user's action caused something- i.e. a progress bar?)

1. FEEDBACK Prompts (Does the dashboard present design dialogues to clarify actions are completed?)

2. ERROR RECOVERY or REVERSAL (Does the dashboard allow users to undo actions?)

2. ERROR PREVENTION (Does the dashboard use clear labels or warnings to prevent user errors?)

3. FLEXIBILITY of System (Does the dashboard have customizable settings or options?)

4. CONSISTENCY of System (Does the dashboard use the same icons/buttons for the same actions?)

5. LEARNABILITY clues (Does the dashboard provide easy-to-follow action steps/directions?)

6. AFFORDANCE or VISUAL cues (Does the dashboard include cues that are easily recognizable, i.e. a clickable link is blue and underlined?)

7. MAPPING or NAVIGATION of user input/output actions (Does the dashboard show you where you are on the page, i.e. a scroll bar?)

8. VISIBILITY of System Components (Does the dashboard have icons or buttons that show available actions for the user?)

8. ACCESSIBILITY of System (Does the dashboard have designs that are accessible to users with disabilities, i.e. visual, hearing impairments? Note: This will be visible via an icon or text blurb regarding support for visual impairment or auditory support.)

9. DOCUMENTATION in System (Does the dashboard keep tab or record of user actions?)

10. SIMPLICITY of System (Does the dashboard exhibit only information that is needed or meaningful?)

11. CONSTRAINTS in System (Does the dashboard use physical/logical limitations to prevent users from certain actions or mistakes?)

Other:

None of these are visible.

QUESTION 4. DECISION SUPPORT

Does my dashboard integrate DECISION SUPPORT mechanisms in the information presented?
Select all that apply.

Yes, Decision Support Visualizations (i.e. Decision trees, Decision Paths, i.e., if # of COVID cases is over 50% then implement vaccine mobile clinic, if [data] then [decision advice])

Yes, Descriptions of Decision-based Outcomes/ Trade-offs (i.e. Description or Prompt of decision trade-offs or considerations, i.e., if # of COVID cases is over 50% then implement vaccine mobile clinic, BUT consider where to remove mobile clinic from or funding to add a new mobile clinic]

No, None of these are visible.

IF YES TO VISUALIZATIONS OR ANALYTICS, REVIEW Q4.1, Q4.2.

IF NO, MOVE TO QUESTION 5.

Q4.1. Check off the Decision Support VISUALIZATIONS you observe in the dashboard:
Select all that apply.

General Decision Guidance (i.e. red light- green light; so if red then data triggers decision prompt to increase vaccine dissemination, if green then no trigger for a decision prompt, so don't increase vaccine dissemination)

Decision Tree (i.e. [IF] a COVID hotspot in Neighborhood A, [THEN] implement a vaccine clinic in Neighborhood A)

Decision Path (Directional arrows or pathways guide to different decision options based on the data)

Other:

Q4.2. Check off the Decision OUTCOMES/ TRADE-OFFS you observe in the dashboard:
Select all that apply.

Decisions define and articulate the outcomes (i.e. [IF] a is implemented, [THEN] schools can reopen)

Decisions define and articulate potential trade-offs (i.e. [IF] a is implemented, [THEN] schools can reopen [BUT] small businesses remain closed.)

Decisions define and articulate uncertainties (i.e. [IF] a is implemented, [THEN] schools can reopen-- [NOTE-- This may lead to higher rates of COVID, or higher rates of Flu, or overburden local clinics].)

Other:

QUESTION 5. EQUITY-BASED DECISIONS

Does my dashboard describe decisions with an EQUITY FOCUS or LENS (i.e. Decisions re: affordability, neighborhood hotspots, etc...)? *Select all that apply.*

Decision options provided in the dashboard are based on community DEMOGRAPHIC data (i.e. population rates, census data, household data)

Decision options provided in the dashboard are based on community ACCESS (i.e., based neighborhood needs assessments and/or hotspots)

Decision options provided in the dashboard are based on community AFFORDABILITY (i.e., based on neighborhood needs assessments and/or hotspots)

Other equity-related decisions:

No, None of these are presented.

QUESTION 6. COMMUNITY-BASED INTERVENTIONS

Does my dashboard present information on any type of INTERVENTION (i.e., community-based interventions, like vaccine clinics; policies, like PTO or workplace policies; funding, like funding opportunities)? *Select all that apply.*

Yes, ANY information on intervention(s) are presented in the dashboard.

No, no intervention(s) are presented in the dashboard.

IF YES TO INFORMATION ON INTERVENTIONS, REVIEW Q6.1, Q6.2.

IF NO, MOVE TO QUESTION 7.

Q6.1. Which INTERVENTION TYPE(S) does the dashboard present or describe?
Select all that apply.

Community Interventions (i.e. Vaccine clinics, Quarantines, Shutdowns, etc...)

Local, State, or National Policies (i.e. A policy to allow all essential workers access to PTO)

Local, State, or National Funding (i.e. Funding opportunities for local health departments)

Other:

Q6.2. Which INTERVENTION SCOPE(S), if any, are described or presented in the dashboard?
Select all that apply.

Schools

Workplaces

Restaurants

Small Businesses

Industry-based (i.e. Factories, Farms, Agriculture, Oil, etc...)

Jurisdictional (City-wide, County-wide, State-wide)

Other:

No, it does not specify.

QUESTION 7. OVERALL RATING + SCORE

Overall, how much do you agree with the statements below:
Select one answer per row.

	Strongly disagree	Somewhat disagree	Neither agree nor disagree	Somewhat agree	Strongly agree
This dashboard presented a satisfactory amount of data on the community it serves, and the needs of the community.	○	○	○	○	○
This dashboard provided a satisfactory amount of information on decision support and guidance.	○	○	○	○	○
This dashboard satisfactorily addressed health equity in the data and analyses.	○	○	○	○	○
This dashboard displayed visualizations, information, and a user interface that was easy to understand and navigate.	○	○	○	○	○

Based on my review of this dashboard and the key takeaways in the DIRE Checklist, I would rate this dashboard a:

→
