# Supplementary material for: Assessing Usefulness of the Dashboard Instrument to Review Equity (DIRE) Checklist to Evaluate Equity in Public Health Dashboards: Reliability Study
Source: JMIR Public Health Surveill. 2025 Dec 4;11:e71094. doi: 10.2196/71094 (PMC12677865; doi:10.2196/71094)
Supplement: Multimedia Appendix 4 [file publichealth-v11-e71094-s004.docx]

**Dashboard Review– Standard Operating Procedure (SOP)**

**M3 Phase**

**PRE-REVIEW:**

1. Open [Dashboard Review- M3 Phase](https://docs.google.com/spreadsheets/d/19Kik2BL_qdXH6wGOfh7A27r0nQGAftQqUBaTbn08ugg/edit#gid=0)
2. Open Qualtrics link: <https://nursingjhu.qualtrics.com/jfe/form/SV_0wxviGwObl4n9LE>
3. Open the Dashboard you wish to review
4. Start Timer manually (via phone or other device)

**DASHBOARD REVIEW**

1. Review the dashboard to briefly familiarize yourself with content (1-2 minutes)
   1. Click through pages, links, other icons present
2. Begin the Qualtrics Checklist Survey
3. Input Dashboard Reference Number (identified in [Dashboard Review- M3 Phase](https://docs.google.com/spreadsheets/u/0/d/19Kik2BL_qdXH6wGOfh7A27r0nQGAftQqUBaTbn08ugg/edit))
4. Input Reviewer Name
5. Start at Q1, review all pages for thorough response to each Question presented in Checklist
   1. For any observations made, or questions identified- make a note in [Dashboard Review- M3 Phase](https://docs.google.com/spreadsheets/u/0/d/19Kik2BL_qdXH6wGOfh7A27r0nQGAftQqUBaTbn08ugg/edit)
6. For Q7, provide your agreement levels per row– although this is a subjective measurement, please notate why you made your choices in [Dashboard Review- M3 Phase](https://docs.google.com/spreadsheets/u/0/d/19Kik2BL_qdXH6wGOfh7A27r0nQGAftQqUBaTbn08ugg/edit)
7. For Q8, provide your overall grade level for the dashboard reviewed– although this is a subjective measurement please notate why you provided the grade you did in [Dashboard Review- M3 Phase](https://docs.google.com/spreadsheets/u/0/d/19Kik2BL_qdXH6wGOfh7A27r0nQGAftQqUBaTbn08ugg/edit)
8. Submit your results to Qualtrics.

**POST-REVIEW**

1. Stop Timer manually
   1. Note the duration of time spent reviewing the selected dashboard
2. Check off the checkbox once review is completed
3. Note the date under “Date Reviewed”
4. Finalize any additional Observations made for selected dashboard
