# Supplementary material for: Assessing Usefulness of the Dashboard Instrument to Review Equity (DIRE) Checklist to Evaluate Equity in Public Health Dashboards: Reliability Study
Source: JMIR Public Health Surveill. 2025 Dec 4;11:e71094. doi: 10.2196/71094 (PMC12677865; doi:10.2196/71094)
Supplement: Multimedia Appendix 5 [file publichealth-v11-e71094-s005.pdf]

Appendix S1- S7:

Figure S1. Category 1 Descriptive Analysis

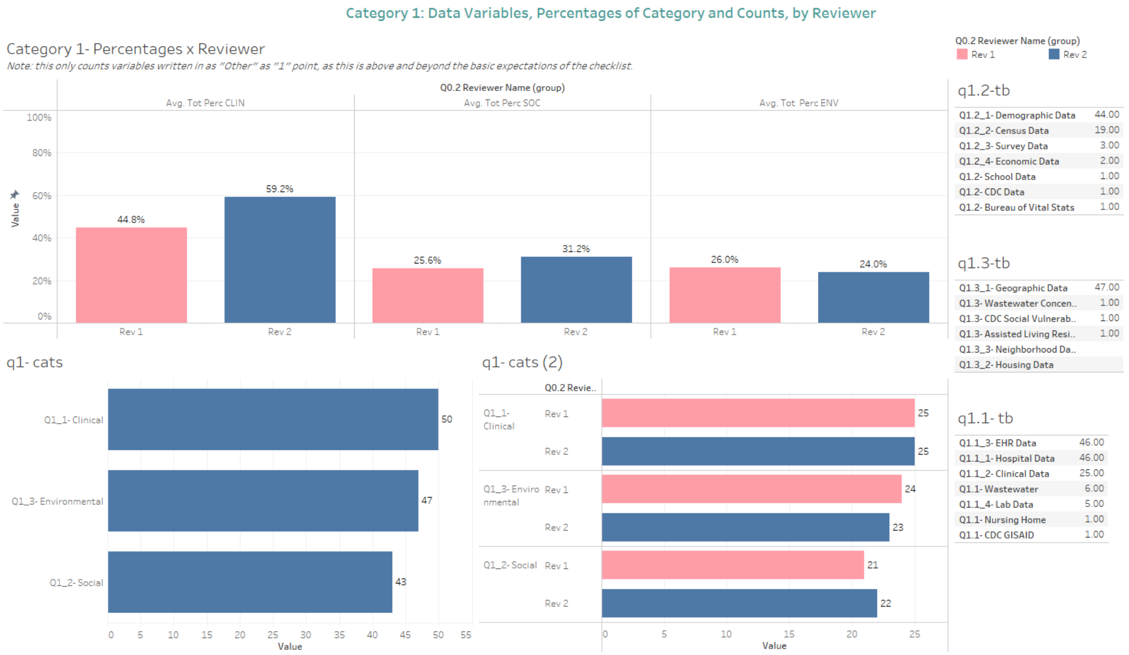

Figure S2. Category 2. Descriptive Analysis

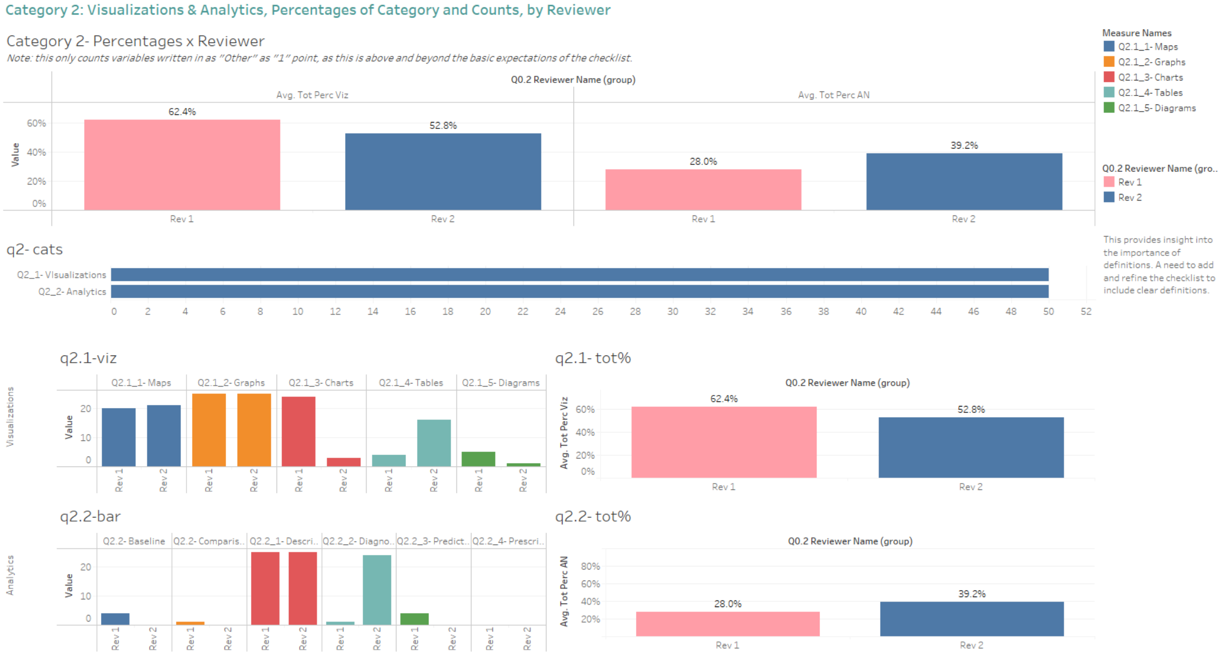

Figure S3. Category 3. Descriptive Analysis

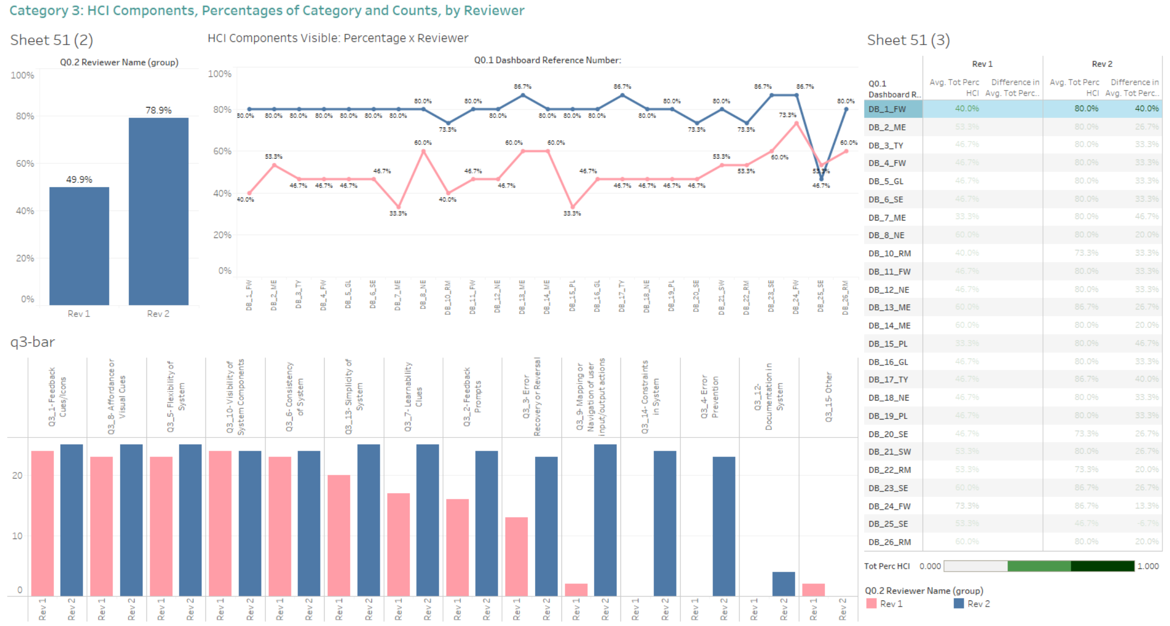

Figure S4. Category 4- Descriptive Analysis

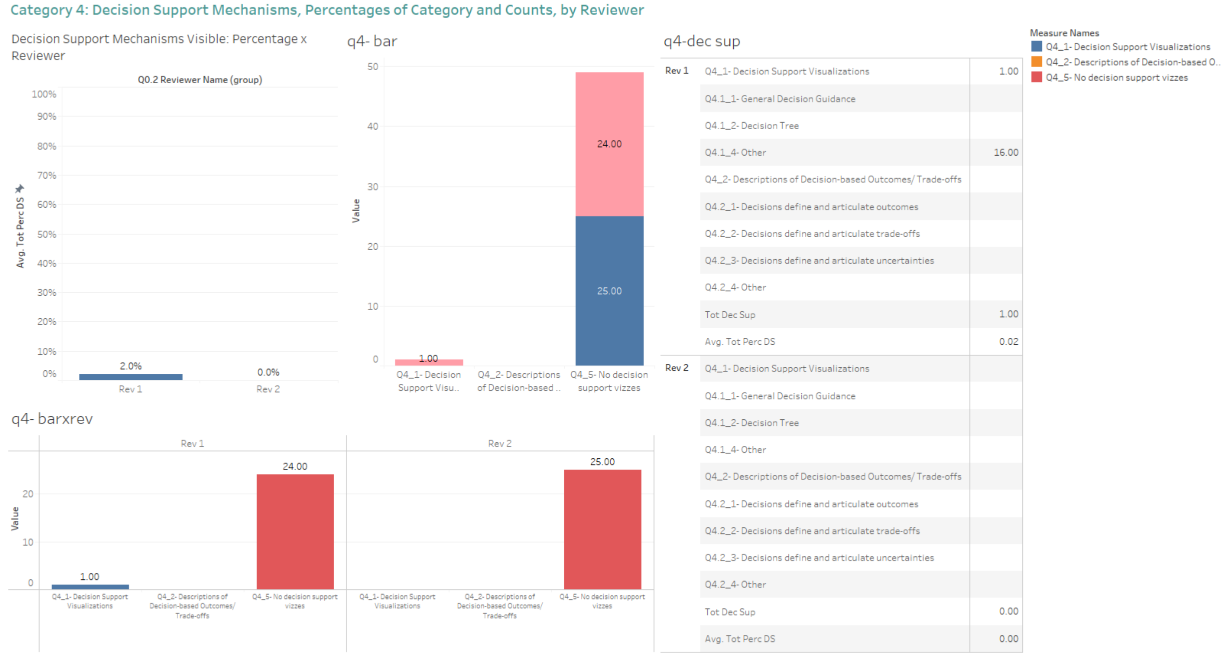

Figure S5. Category 5- Descriptive Analysis

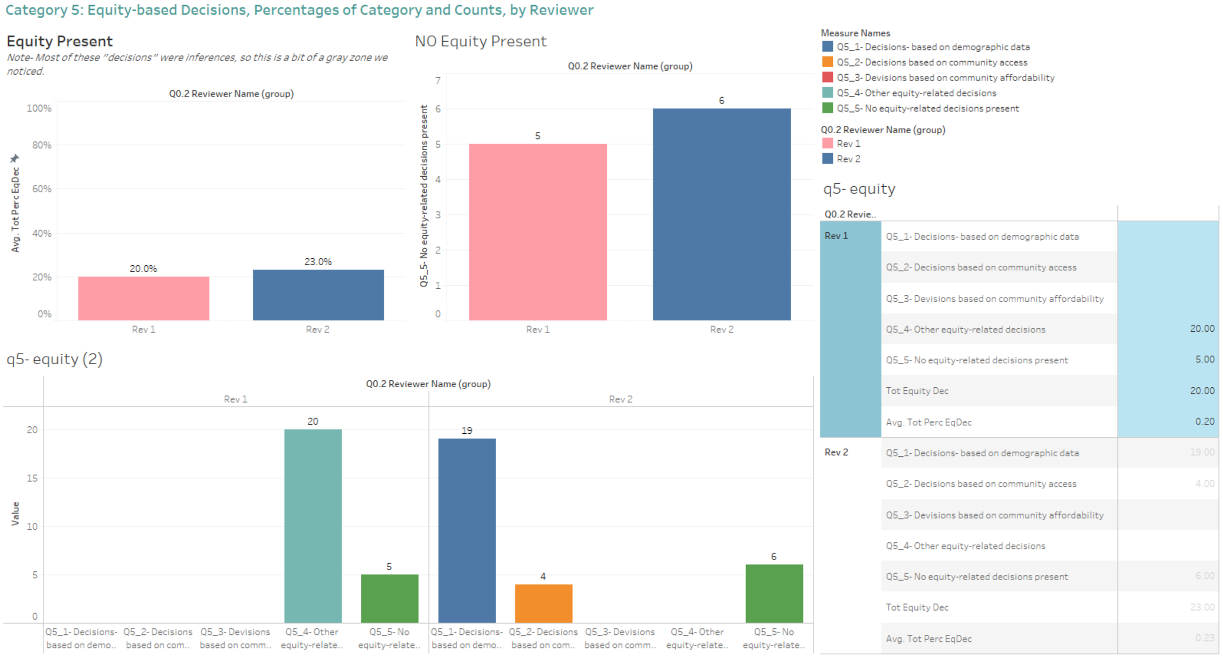

Figure S6. Category 6- Descriptive Analysis

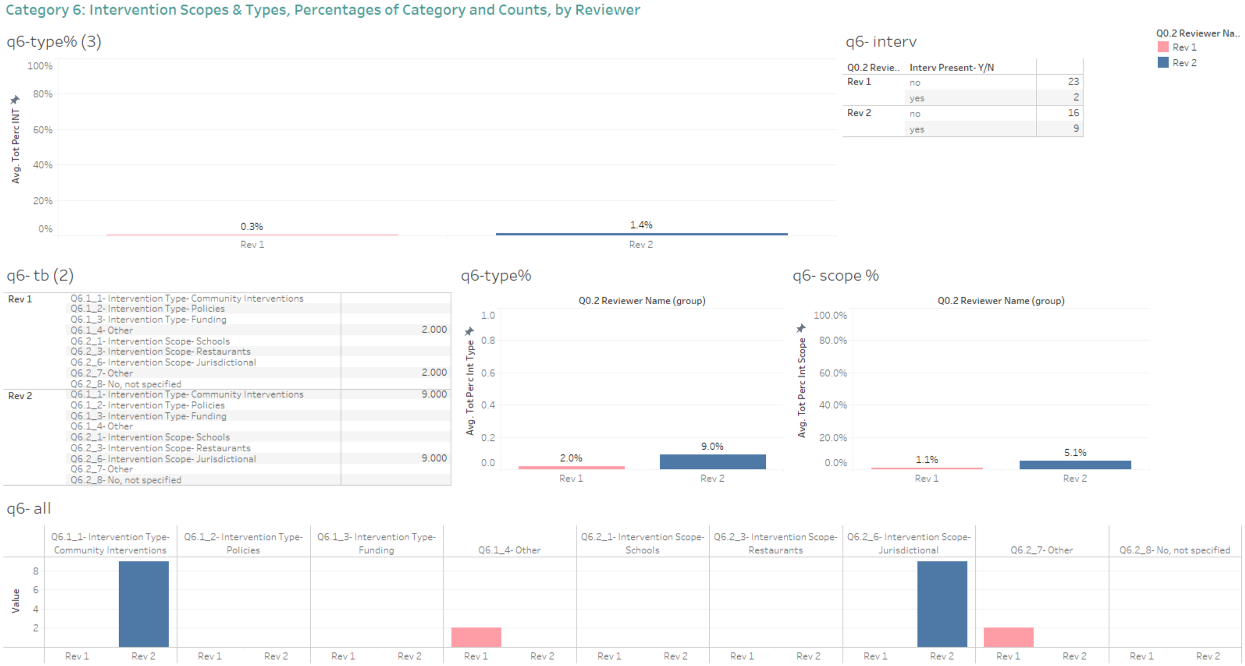

Figure S7. Overall Scores (Qualtrics and Reviewer Score)- Descriptive Analysis

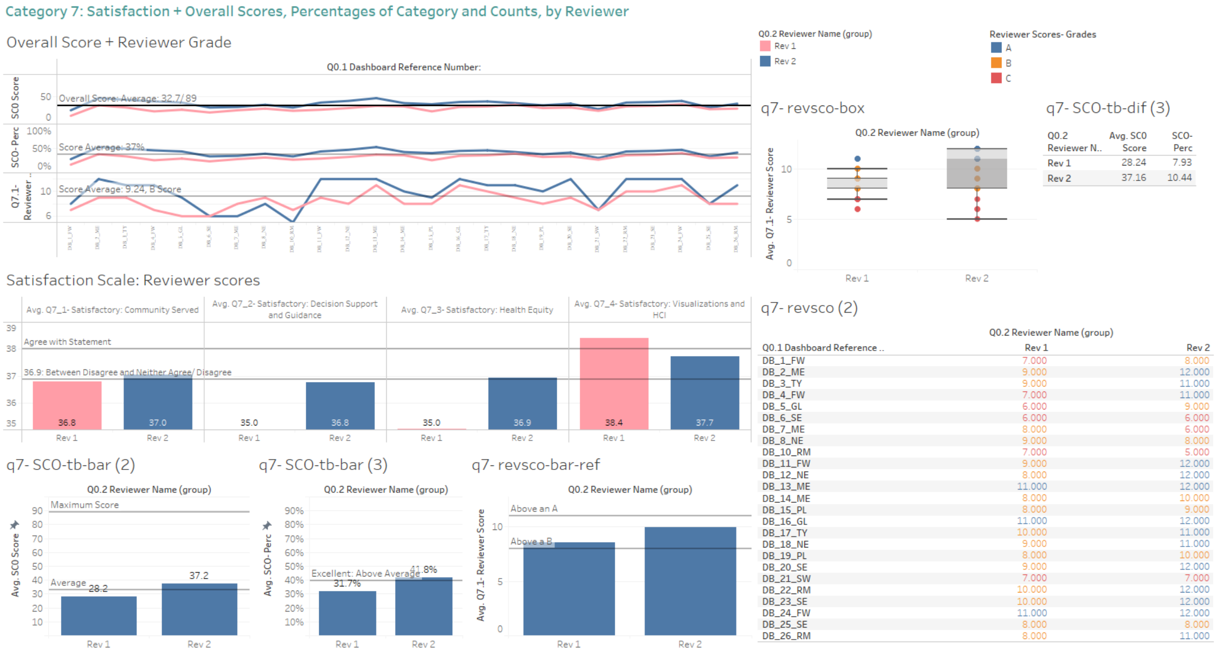

Q0.2 Reviewer N..	Avg. SCO Score	SCO-Perc
Rev 1	28.24	7.93
Rev 2	37.16	10.44

Q0.1 Dashboard Reference ..	Q0.2 Reviewer Name (group) Rev 1	Rev 2
DB_1_FW	7.000	8.000
DB_2_ME	9.000	12.000
DB_3_TY	9.000	11.000
DB_4_FW	7.000	11.000
DB_5_GL	6.000	9.000
DB_6_SE	6.000	6.000
DB_7_ME	8.000	6.000
DB_8_NE	9.000	8.000
DB_10_RM	7.000	5.000
DB_11_FW	9.000	12.000
DB_12_NE	8.000	12.000
DB_13_ME	11.000	12.000
DB_14_ME	8.000	10.000
DB_15_PL	8.000	9.000
DB_16_GL	11.000	12.000
DB_17_TY	10.000	11.000
DB_18_NE	9.000	11.000
DB_19_PL	8.000	10.000
DB_20_SE	9.000	12.000
DB_21_SW	7.000	7.000
DB_22_RM	10.000	12.000
DB_23_SE	10.000	12.000
DB_24_FW	11.000	12.000
DB_25_SE	8.000	8.000
DB_26_RM	8.000	11.000
