# Supplementary figures and images for: Assessing Usefulness of the Dashboard Instrument to Review Equity (DIRE) Checklist to Evaluate Equity in Public Health Dashboards: Reliability Study
Source: JMIR Public Health Surveill. 2025 Dec 4;11:e71094. doi: 10.2196/71094 (PMC12677865; doi:10.2196/71094)

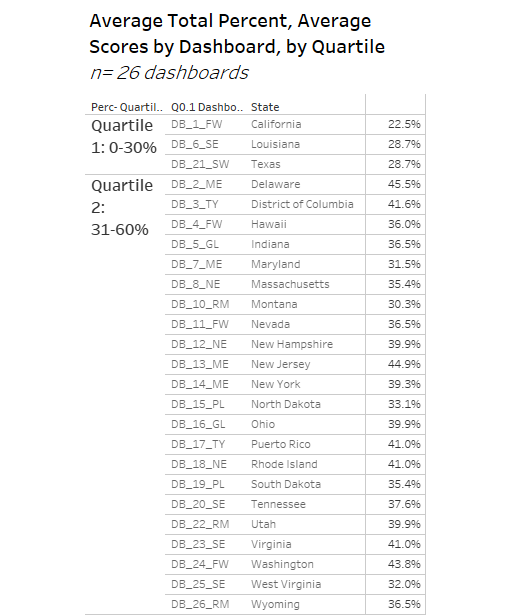

Supplement: Multimedia Appendix 6 [file publichealth-v11-e71094-s006.png]
