# Supplementary material for: Assessing Usefulness of the Dashboard Instrument to Review Equity (DIRE) Checklist to Evaluate Equity in Public Health Dashboards: Reliability Study
Source: JMIR Public Health Surveill. 2025 Dec 4;11:e71094. doi: 10.2196/71094 (PMC12677865; doi:10.2196/71094)
Supplement: Multimedia Appendix 7 [file publichealth-v11-e71094-s007.docx]

APPENDIX G: Thematic Analysis Methods and Results

Reviewers entered their comments as textual notes in a spreadsheet during the user testing process. These notes were “explored” for potential common themes; however, a full thematic analysis was not feasible due to the limited textual information.

***Methods***

The notes were coded by each reviewer for recurring themes related to dashboard design, checklist usability, and suggestions for improvement. Upon completion of the coding process, a limited thematic analysis was conducted to review findings and explore potential patterns or themes.

***Results***

The thematic analysis categorized the wide range of topics frequently referenced in reviewer notes, with “Data” being the most mentioned theme (117 mentions), followed by “Equity” (42 mentions) and “Decision Support” (26 mentions). Other themes, such as “Dashboard”, “Demographic Data”, and “Completeness”, also emerged as notable themes, but were at much lower frequencies. These themes primarily reflected elements that were already part of the dashboard evaluation process and closely aligned with the DIRE checklist in scoring.

The variability in reviewers’ notes, ranging from comments on areas that needed improvement in specific dashboards to updates for future iterations of the DIRE checklist, showcased that these themes did not substantially inform the dashboard scoring or checklist evaluation. Instead, these comments served as supplementary observations related to dashboard content and function or the checklist itself and potential ways to refine it further.

Given this context, the thematic analysis was not utilized to adjust scoring but was rather referenced as part of the broader process of evaluation and checklist refinement. “Data” and “Equity”, as dominant themes, underscore the key areas where dashboards either met (i.e., data was the highest quantitative category) or failed to meet (i.e., equity was one of the lowest categories) reviewer expectations but did not necessarily impact the quantitative results.
